# Neuroprotective effect of memantine on serum S100-B levels after on-pump coronary artery bypass graft surgery: A randomized clinical trial

**DOI:** 10.22088/cjim.13.2.412

**Published:** 2022

**Authors:** Shervin Ziabakhsh Tabary, Parham Ziabakhsh Tabary, Alireza Sanei Motlagh

**Affiliations:** 1 Department of Cardiac Surgery, Cardiovascular Research Center Mazandaran University of Medical Sciences, Sari, Iran; 2 Faculty of Specialized Veterinary Sciences, Islamic Azad University, Science and Research Branch, Tehran, Iran; 3 Mazandaran University of Medical Sciences, Sari, Iran

**Keywords:** Serum S100-B, Memantine, Cardiopulmonary bypass, Coronary artery bypass surgery

## Abstract

**Background:**

Brain injury is one of the complications of open heart surgery. Glutamate plays a key role in this process. In this study, we evaluated the neuroprotective effect of memantine as NMDAR inhibitor in patients undergoing on-pump coronary artery bypass graft surgery (CABG).

**Methods:**

From July 2019 to May 2020, thirty-four consecutive patients selected for elective isolated on-pump coronary artery bypass graft surgery (CABG) enrolled in the trial. Patients were randomly assigned into two groups; memantine and the control group. For the memantine group, 10 mg of memantine twice daily was administered at least 72 h before surgery. Venous blood samples were collected before surgery (T1), at the end of cardiopulmonary bypass (CPB) (T2), 6h and 24h after CPB (T3 and T4). Serum concentration of S100-B was measured by enzyme-linked immunosorbent assay (ELISA) technique.

**Results:**

Serum S100-B increased during CPB with a peak plasma concentration at the termination of CPB. Then it gradually decreased during the first 24 hours in both groups (*P*=0.001). The mean S100-B levels were significantly lower in the memantine group compared to the control group at the termination of CPB (0.863±0.203 μg/l vs 1.117±0.304 μg/l), at 6 hours post-CPB (0.731±0.168 μg/l vs 0.938±0.206 μg/l), and 24 hours post-CPB (0.595±0.189 μg/l vs 0.852±0.227 μg/l), respectively (*P*=0.023). The mean level of serum S100-B in memantine group was about 0.19 μg/l less than the control group during the study (CI, 0.07 to 0.30; *P*=0.001). One (6.2%) patient in the control group had post-operative left arm paresthesia

**Conclusion:**

Administration of memantine before on-pump CABG can attenuate the post-operative concentrations of serum S100-B, which may reduce cerebral damage during surgery.

Cerebral injury is one of the complications of open heart surgery, during which there is a possibility of embolism, tissue hypoperfusion, hyperglycemia and anemia. These factors lead to ischemia of brain tissue (1). Complications including stroke (1.5–5.2%), encephalopathy (8.4–32%), and cognitive dysfunction (20–30%) are reported during the post-operative period after on-pump cardiac surgeries (2-5). Glutamate is a neurotransmitter that plays a key role in brain function. At the time of brain injury, its concentration reaches 50-folds the baseline level, which can lead to neuronal death.

Glutamate affects the learning and cognition process by acting on NMDA receptors. Increase in glutamate concentration leads to an increase in neuronal excitability through these receptors which in turn trigger the process of neuronal apoptosis (6-8). S100-B protein is normally expressed in astrocytes and specific neuronal populations. It involves in regulating cell function and apoptosis. During neuronal damage, this protein is released from the cell and can be spread and measured in plasma (9). The use of cardiopulmonary bypass (CPB) in cardiac surgery can lead to long-term neurological events. Previous studies have shown that S100-B levels increase after cardiopulmonary bypass and then gradually decrease up to 48 hours thereafter. It has also been shown that S100-B concentrations greater than 1.5 μg/l after CPB can be associated with impaired memory function, neurological disorders, and longer duration of stay at hospital (10). Among the strategies defined to prevent brain damage during open heart surgery, NMDA receptor antagonists are somewhat known. Memantine as a non-competitive NMDAR inhibitor prevents neuronal excitability and used to treat Alzheimer's disease and dementia (11, 12). Memantine is able to inhibit the long-term influx of Ca2+ ions, especially from the extra synaptic receptors that form the basis of neuronal excitability. However, the low affinity, non-competitive nature, and rapid-acting kinetics of memantine at the NMDA receptor channels maintain the receptor function at synapses so that receptors can still be stimulated by glutamate (13, 14). Due to the importance and significant percentage of brain injury after open heart surgery, the aim of this study was to investigate the effect of memantine as a NMDA receptors antagonist during on-pump CABG and evaluate its effect on post-operative serum S100-B levels.

## Methods

**Patients: **This study was designed as a single institution, double-blinded randomized clinical trial. From July 2019 to May 2020, thirty-four consecutive patients selected for elective isolated on-pump coronary artery bypass graft surgery (CABG) enrolled in the trial. Patients who underwent emergency CABG, those with myocardial infarction (MI) within 72 h before the surgery, ipsilateral carotid artery stenosis more than 70%, bilateral carotid artery stenosis more than 50%, and those with the history of acute renal failure (stage 4/5), creatinine clearance below 30 ml/min, moderate to severe hepatic impairment (ChildPugh B/C), auto-immune disease, dementia, psychiatric illness, stroke, and seizure were excluded. Demographic data (including age, sex, and health status prior to surgery), history of diabetes mellitus, hypertension, and dyslipidemia were collected. 

**Intervention and surgical technique: **For all enrolled patients, blood samples were collected just 4 days before operation (T1). Patients were randomly assigned into memantine (10 mg under brand name Ebixa, Lundbeck, Denmark) and the control group. For the memantine group, 10 mg of memantine (tab Ebixa 10 mg, Lundbeck, Denmark) twice daily administered at least 72 h before surgery, and continued for the first 24 h after surgery. Patients in control group received placebo before surgery. All bypass grafts surgeries were performed via cardiopulmonary bypass (CPB) and operated by the same surgeon under the same condition. Balanced anesthesia was inducted with midazolam, sufentanil, and pancuronium. Patients were mechanically ventilated with a mixture of oxygen and isoflurane via endotracheal tube. After median sternotomy, the left internal mammary artery harvested as pedicled fashion for the left anterior descending artery and saphenous vein grafts was prepared for other coronary arteries. Following aortic and right atrial cannulation, CPB was conducted with moderate hypothermia and simultaneous antegrade and retrograde cardioplegia. All bypass grafts were performed on non-beating heart. 

**The S100-B analysis: **Venous blood samples were collected at the end of CPB (T2), 6 h (T3), and 24 h post CPB (T4). Then, serum extraction was carried out in accordance with related documents and samples kept at -70℃. Serum concentration of S100-B protein were measured by enzyme-linked immunosorbent assay (ELISA) technique (Human S100B / S100 Beta (Sandwich ELISA) ELISA Kit, LSBio, Inc., Seattle, WA, USA). The detection limit of this technique is 0.156 μg/l. 

**Figure 1:**
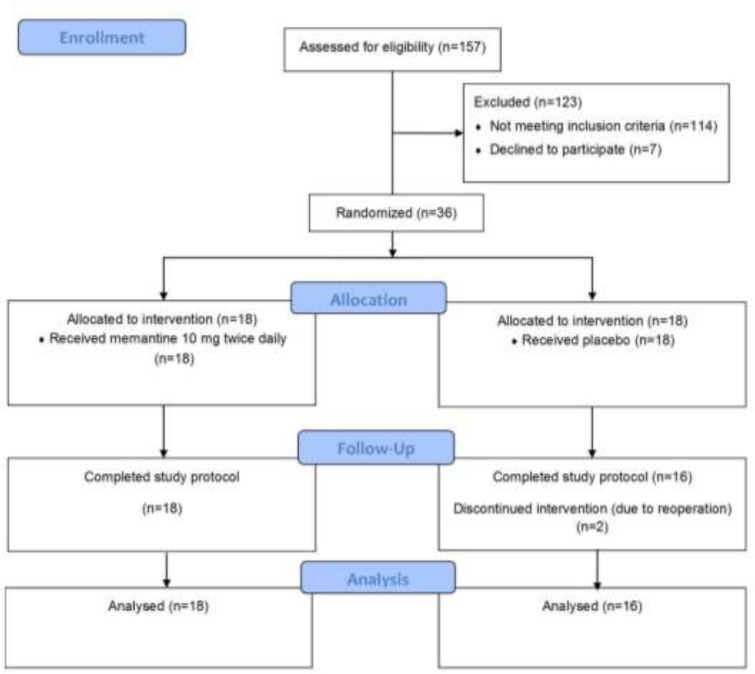
CONSORT flow diagram of enrolled patients through the trial

**Trial registration and ethics: **Our study was conducted according to the Helsinki Declaration. The study protocol was approved by the Research Ethics Committee of Mazandaran University of Medical Sciences, and Mazandaran Cardiovascular Research Center (IR.MAZUMS.REC.1398.333). Furthermore, the trial was registered in the Iranian Registry of Clinical Trials (registry number: IRCT20200502047276N1). All participants gave written informed consent before intervention. The CONSORT (Consolidated Standards of Reporting Trials) recommendations for reporting randomized controlled clinical trials were followed (figure 1). 

**Statistical analysis: **The numerical variables were expressed as mean and standard deviation (SD). The data were compared between the two groups using chi-square test, Fisher's exact test, or Mann-Whitney U tests. Longitudinal changes in the parameters were compared between the two groups by general linear model repeated-measurement analysis of variance (ANOVA). Generalized estimating equation (GEE) was used to estimate the correlation between parameters and outcomes. All the statistical analyses were performed using the SPSS Version 24.0 program (IBM corp., Chicago IL, USA). A p>0.05 was considered statistically significant.

## Results

A total of 34 patients who underwent on-pump CABG and met the inclusion criteria were enrolled in this study, with 18 in memantine group and 16 in the control group. A summary of patients' characteristics is given in table 1. There were 22 (64.7%) males and 12(35.3%) females with the mean age of 63.7±8.1 (mean±standard deviation). There were no significant differences between the two groups regarding age and gender distribution. Fifteen (44.1%) patients had a history of diabetes, twenty-five (73.5%) patients had hypertension, and dyslipidemia was reported in nine (26.5%) patients. There were also no significant differences between two groups regarding these variables. 

**Figure 2:**
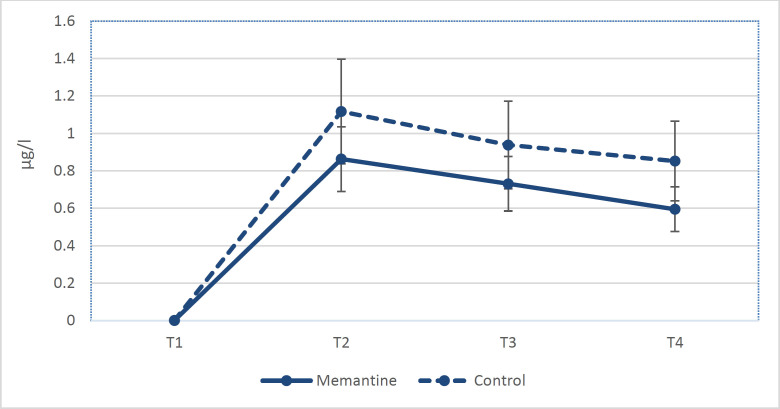
Estimated marginal means of serum S100-B concentrations

The mean length of cardiopulmonary bypass was 58.6±14.7 min for memantine group versus 58.2±16.7 min for control group (*P*=0.59). The mean cross-clamp time for memantine and control group were 27.6±7.1 min and 31.0±11.1 min, respectively (*P*=0.41). There was no significant difference between two groups in terms of total grafts used for CABG. Protein S100-B serum concentrations at the termination of CPB, 6hr, and 24hr post-CPB are shown in table 2. For both groups, S100-B serum concentrations reached the maximum level at the termination of CPB (0.863±0.203 μg/l for memantine group vs 1.117±0.304 μg/l for control group, *P*=0.023). Then the concentration of S100-B slightly decreased during first 24 hours in each group (*P*=0.001). The mean post-CPB S100-B levels were significantly lower in the memantine group rather than the control group (0.731±0.168 μg/l vs 0.938±0.206 μg/l at 6hr post-CPB, and 0.595±0.189 μg/l vs 0.852±0.227 μg/l at 24hr post-CPB, respectively, *P*=0.023) (Figure 2). Estimating the correlation of parameters with serum S100-B by GEE model revealed that the mean level of serum S100-B in memantine group was about 0.19 μg/l less than control group during the study (CI, 0.07 to 0.30; *P*=0.001) (table 3). There were no pre-/post-operative complications with regard to memantine administration. All patients extubated at 4 to 6 hours after surgery. One (6.2%) patient in control group had post-operative left arm paresthesia. The S100-B concentration in this patient was significantly higher than the other participants, so that the S100-B levels were 1.8 μg/l, 1.33 μg/l, and 1.29 μg/l at the termination of CPB, 6 hours, and 24 hours post-CPB, respectively. Further imaging studies confirmed the ischemic cerebrovascular accident (CVA) in the middle cerebral artery territory. Based-on the post-operative visits and follow-ups, no 30-days mortality was reported for any patient. 

**Table 1:** Patients' characteristics comparing memantine versus control group during on-pump CABG

**Parameter**	**Memantine** **(n=18)**	**Control** **(n=16)**	***P*** **-value** ^*^
Male sex, n (%)	12 (66.7%)	10 (62.5%)	0.80
Age, mean (SD) (years)	63.8 (8.8)	63.6 (7.6)	0.34
Diabetes, n (%)	7 (38.9%)	8 (50.0%)	0.52
Hypertension, n (%)	13 (72.2%)	12 (75.0%)	0.58
Dyslipidemia, n (%)	5 (27.8%)	4 (25.0%)	0.58
≥ 3 grafts, n (%)	17 (94.4%)	14 (87.5%)	0.59
CPB time, mean (SD) (min)	58.6 (14.7)	58.2 (16.7)	0.59
Cross-clamp time, mean (SD) (min)	27.6 (7.1)	31.0 (11.1)	0.41
Major neurologic event (post-op), n (%)	0 (0)	1 (6.2%)	-

^*^ *P-*values are generated by chi-squared test and Fisher's exact test

SD: standard deviation; CPB: cardiopulmonary bypass

**Table 2:** Protein S100-B plasma concentrations in memantine versus control group

	**Pre-op** **(T1)**	**At termination of CPB** **(T2)**	**6hr post-CPB** **(T3)**	**24hr post-CPB** **(T4)**	***P*** **-value** ^*^
Memantine	0.000	0.863 ± 0.203	0.731 ± 0.168	0.595 ± 0.189	0.001
Control	0.000	1.117 ± 0.304	0.938 ± 0.206	0.852 ± 0.227	0.001

^*^Statistics are generated by repeated Measurement ANOVA test. Correlation of S100-B determinations within two groups: pre-op (T1) vs at the termination of CPB (T2) vs 6hr post-CPB (T3) vs 24hr post CPB (T4) (*p* = 0.001). Between groups analysis showed that the mean post-CPB S100-B concentrations were significantly lower in the memantine group (*p* = 0.023). Data presented as mean ± standard deviation (μg/l); pre-op: preoperatively; CPB: cardiopulmonary bypass

**Table 3:** Estimating the correlation of parameters with serum S100-B by GEE model

**S100-B**	**estimate**	**Standard error**	**95% CI**		**Z**	**Pr > |Z|**
Group	0.1903	0.0579	0.0766	0.3039	3.28	0.001
Sex	-0.0900	0.0523	-0.1927	0.0126	-1.72	0.086
Age	0.0051	0.0054	-0.0054	0.0157	0.95	0.341
CC time	-0.0058	0.0072	-0.0199	0.0082	-0.81	0.419
CPB time	-0.0021	0.0040	-0.0101	0.0057	-0.54	0.592
Total graft	0.0581	0.1083	-0.1541	0.2704	0.54	0.591

CC: cross-clamp; CPB: cardiopulmonary bypass; CI: confidence interval; Pr > |Z|: p-value; Z: z-statistic

Serum S100-B concentrations among study groups measured before surgery (T1), immediately after CPB termination (T2), at 6 hours (T3), and 24 hours (T4) after CPB. A peak of S100-B concentration is noted in both groups at the termination of CPB. Post-operative S100-B levels were markedly lower in memantine group (p = 0.023)

## Discussion

The present study revealed that preoperative administration of memantine, followed by its administration during first 24 hours after surgery attenuates S100-B increase in patients that underwent on-pump CABG. Brain injury is known as one of the complications of cardiac surgery. The clinically significant injuries will be associated with higher mortality and morbidity rates which lead to loss of the health care resources. The S100-B protein is correlated with cerebral injury even at the cellular level. Higher levels of plasma S100-B can indicate extensive ischemic area of the brain (15). In previous studies, it was mentioned that S100-B markedly is released during CPB. The maximum levels of serum S100-B were seen at the termination of CPB. This phenomenon is probably due to the use of cardiotomy suction during on pump surgeries, which the blood is contaminated with the vast source of S100-B expressing cells from mediastinum. After that, S100-B level gradually decreases where it may not be detectable after 48 hours (10, 16). Neuron-specific enolase (NSE) is another biomarker of brain injury. Ozkisacik et al. (17) reported that the measures of NSE in patients without prior neurological deficit that underwent on-pump cardiac surgeries were not significantly different during pre-operative and post-operative course. They also mentioned that the central nervous system damage due to CPB may not be to the extent causing a significant rise in serum NSE levels. In our study, S100-B increases during CPB in both groups. It reached the maximum concentration at the termination of CPB, and gradually decreased during the first 24 hours after CPB. However, the mean serum concentration of S100-B was about 0.19 μg/l lower in memantine group. This reveals the potential effect of memantine as NMDAR inhibitor to prevent an increase in S100-B serum levels during on-pump CABG.

Memantine acts by the non-competitive blocking of NMDA receptors on the surface of neurons. This drug prevents neuronal excitability during ischemic or degenerative processes, a condition similar to that of on-pump cardiac surgeries (18, 19). In our study, no major neurologic event was noted in memantine group. However, one patient in control group had left arm paresthesia after surgery in which further imaging studies confirmed the ischemic source in the brain. The S100-B level was 1.8 μg/l at the termination of CPB for this patient. As Sevenmarker *et al.* mentioned earlier, S100-B concentrations greater than 1.5 μg/l after CPB are associated with neurological events and memory dysfunction (10). Our study had some limitations. One of them was the small population. From the entire population of patients who referred to the heart center, we selected patient candidates for isolated on-pump CABG for enrollment. Another limitation was short follow-up duration. However, our findings showed significant reduction in post-CPB S100-B measures among patients in memantine group. 

In conclusion** t**he present study demonstrates that pre-operative administration of memantine in patients undergoing on-pump CABG can attenuate serum S100-B increase during the cardiopulmonary bypass. Its administration is safe and no adverse effect was noted for it. 
